# Increased Basal Activity Is a Key Determinant in the Severity of Human Skeletal Dysplasia Caused by *TRPV4* Mutations

**DOI:** 10.1371/journal.pone.0019533

**Published:** 2011-05-05

**Authors:** Stephen Loukin, Zhenwei Su, Ching Kung

**Affiliations:** 1 Laboratory of Molecular Biology, University of Wisconsin–Madison, Madison, Wisconsin, United States of America; 2 Department of Genetics, University of Wisconsin–Madison, Madison, Wisconsin, United States of America; VIB & Katholieke Universiteit Leuven, Belgium

## Abstract

TRPV4 is a mechanically activated Ca^2+^-passing channel implicated in the sensing of forces, including those acting on bones. To date, 33 mutations are known to affect human bone development to different extents. The spectrum of these skeletal dysplasias (SD) ranges from dominantly inherited mild brachylomia (BO) to neonatal lethal forms of metatropic dysplasia (MD). Complexities of the results from fluorescence and electrophysiological studies have led to questions on whether channel activity is a good predictor of disease severity. Here we report on a systematic examination of 14 *TRPV4* mutant alleles covering the entire SD spectrum. Expressed in *Xenopus* oocyte and without any stimulation, the wild-type channel had a ∼1% open probability (Po) while those of most of the lethal MD channels approached 100%. All mutant channels had higher basal open probabilities, which limited their further increase by agonist or hypotonicity. The magnitude of this limitation revealed a clear correlation between the degree of over-activity (the molecular phenotype) and the severity of the disease over the entire spectrum (the biological phenotype). Thus, while other factors are at play, our results are consistent with the increased TRPV4 basal activity being a critical determinant of the severity of skeletal dysplasia. We discuss how the channel over-activity may lead to the “gain-of-function” phenotype and speculate that the function of wild-type TRPV4 may be secondary in normal bone development but crucial in an acute process such as fracture repair in the adult.

## Introduction

Mutations in the transient receptor potential cation channel TRPV4 have been found to cause many different forms of skeletal dysplasias (SD) [1], [2], [3], [4], [5]. These range from mild autosomal-dominant brachyolmia (BO) [5], diagnosed by a shortened spine with characteristic vertebral defects but only minor defects in the long bones [6] to metatropic dysplasia (MD) [2], [3], [4], characterized by more prominent spine defects as well as pronounced abnormalities in articular skeleton resulting in short dumbbell-shaped long bones [7], which leads to prenatal lethality in its severest forms [8]. Clinically distinguishable spondylometaphyseal dysplasia, Kozlowski type (SMDK) presents defects intermediate in severity [9]. Most recently, *TRPV4* mutations were also found to cause two other clinically distinguishable SD's: spondylo-epiphyseal dysplasia Maroteaux type (SDEM-PM2) and the rare parasymetric dysplasia (PD) [1]. That *TRPV4* mutation causes SD was initially discovered through a pedigree analysis of autosomal dominant BO patients from two familial lines followed by position cloning [5]. Subsequent analysis of 59 genetically diverse and mostly unrelated MD and SMDK patients found that all but one resulted from dominant TRPV4 mutations [1], [2], [3], [4], [5]. In most cases, mutations occurred *de novo*, often appearing to arise from gonadal mosaicism in the parent [10]. Four mutations in two of the six N-terminal ankyrin repeats of TRPV4 have also recently been shown to cause a spectrum of late-onset neuromuscular diseases but not skeletal dysplasia [11], [12], [13], indicating that SD and these neuromuscular diseases arise by different mechanisms [14]. This report covers only the mutations causing skeletal dysplasias and is not concerned with the four alleles causing neuromuscular disease nor the P19S polymorphism, which loosely correlate with serum hyponatremia [15] and chronic obstructive pulmonary disease [16].

The mechanism by which TRPV4 mutation causes skeletal dysplasia is unclear. TRPV4 is a polymodal Ca^2+^-permeable cation channel of 871 AA [17], [18]. It shows a very prominent outward rectification, rarely opening upon hyperpolarization. Mutational analysis suggests that outward rectification is governed by a gating mechanism independent of the main intracellular gate [19]. It also shows a rectification in its unitary conductance, being 98-pS outward and 45-pS inward [20]. Like other TRP channels, TRPV4 is polymodal, activated by mild heat [21], by the intrinsic agonists arachidonic acid and its metabolite 5′,6′-epoxyeicosatrienoic acid [22], or extrinsic agonists such as 4α-phorbol 12,13-didecanoate (4α PDD) [23] and other phorbol derivatives [24], Bisandrographolide [25] and GSK1016790A (GSK) [26]. TRPV4 is also regulated by Ca^2+^ [27], [28], in part through calmodulin [29], as well as by other intracellular messengers [30], [31], [32], [33], [34]. TRPV4 has been implicated in various force- or geometry-sensing functions including systemic osmolarity regulation [35], viscosity-coupled epithelial ciliary activity [36], strain-induced endothelial cell reorientation [37], keratinocyte cell-volume regulation [38], and load sensing of bone [39]. Hypotonic [17], [18] or shear stress [40] activates TRPV4 but the molecular mechanisms are controversial. It has been proposed that mechanical activation was indirect, occurring through mechanical activation of phospholipaseA2 [41]. Recently, Loukin *et al.* (2010) [20] showed that TRPV4 is activated directly by stretch force in excised membrane patches. TRPV4's direct molecular mechanosensitivity provides the simplest interpretation for its roles in force-related physiologies.

While broadly expressed, TRPV4 promotes terminal differentiation and inhibits apoptosis of bone-absorbing osteoclast through a Ca^2+^-dependent signaling pathway [42]. Yet, in stark contrast to the prominent pathologies caused by the mutations studied here, a complete loss of TRPV4 activity has only minor and late-onset effects on bone development. *trpv4^−/−^* knockout is not lethal and the mice display only superficial skeletal defects. One report found a 67% increase in density of femoral trabecula in the tomograms of adult, but not immature *trpv4^−/−^* mice [42]. A concurrent report, however, did not observe such a chronic increase in trabecular density. Instead, *trpv4^−/−^* mice were found to lack “unloading osteoporosis”, in which wild-type bones lose density when they are deprived of the weights they normally bear [39]. In both studies, increased osteoclast populations paralleled decreased bone densities. In a recent report, TRPV4 knockout resulted in the enlargement of certain joint bones consistent with osteoarthritis, but only after 9 months and only in male mice [43].

Thirty-three unique TRPV4 mutations have been found to cause SD to date [1], [2], [3], [4], [5]. They occur throughout the protein, most commonly between the predicted 4th and 5th membrane-spanning domains or near the calmodulin-binding domain in the cytoplasmic C terminus. Whereas most of the observed mutations occurred in only one or occasionally two cases, R594H were found to recur in 17 and P799L in 15 unrelated individuals [1], [2], [3], [4]. In the initial reports the correlation between clinical severity and genotype was absolute, with all R594H individuals having SMDK and all P799Ls having MD. Subsequently, 2 of the 6 SDEM-PM2 cases were found to be due to P799L mutation and the sole PD [1], which is clinically more severe than SMDK, was due to R594H, indicating that other factors besides *TRPV4* allelism can influence disease outcome.

The molecular phenotypes of these mutant channels examined to date are puzzling. Using Fura-2 fluorescence to gauge the Ca^2+^ through TRPV4, the absolute responses to the synthetic agonist 4αPDD of I331F, P799L [3] and D333G were larger than that of the wild type, but those of R594H and A716S were smaller [4]. The responses to the natural agonist arachidonic acid and to hypotonicity were smaller in D333G, R594H and A716S [4]. Because it is technically demanding, direct electrophysiological examinations were understandably sparse. Mutant alleles were expressed in HEK293 cells and their whole-cell current registered [3], [4], [5]. Basal currents were found to increase in the cases of R616Q, V620I [5], D333G, R594H [4] I331F and P799L [3]. A716S, however, resulted in current densities indistinguishable from those of wild type [4]. The complexity of the molecular phenotypes as well as the fact that mutations are scattered throughout the protein has led to questions as to whether increase in channel activity (“gain-of-function” molecular phenotype) determines the disease outcome or whether a different dominant negative mechanism may be operational [1].

Here we report on a systematic electrophysiological analysis of 14 different dysplasia-causing mutants chosen for their allelic and clinical diversity. A strong correlation was observed between clinical severity and an increase in the deduced basal open probabilities. Our results are consistent with the chronic basal open probability of the mutant TRPV4 being a key, though probably not the only, determinant of the severity of skeletal dysplasia in human.

## Results

### Mutant Channels Produce Higher Current Densities

**Figure 1:**
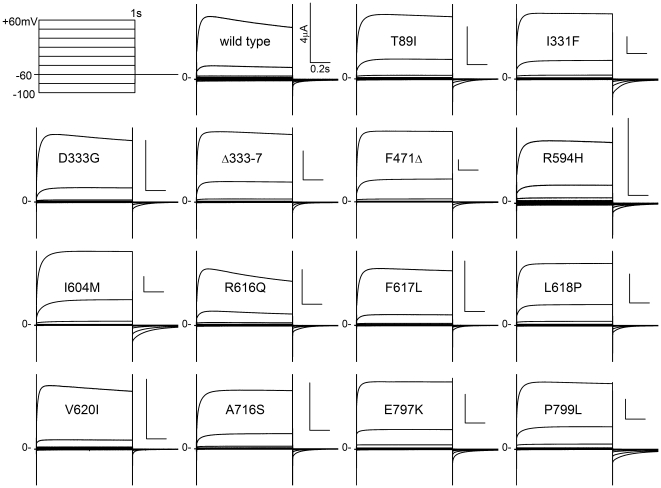
All fourteen mutant *TRPV4* cRNAs tested generated robust currents in Xenopus oocytes. Two to four days after the injection of 22 ng of wild-type or lesser amounts of mutant cRNAs, oocytes were examined with a two-electrode voltage clamp, held at −60 mV and tested every 5 sec between −100 and +60 mV for 1 sec. All mutants retained strong rectification against steady-state inward current. See text. All calibration bars are 4 µA×0.2 sec.

Of the 33 unique TRPV4 mutations so far found to cause SD [1], [2], [3], [4], [5], we chose 14 to represent different mutational locations and to cover the entire range of clinical severity they produce. We have shown previously that rat-TRPV4 cRNA generates a robust microampere current in *Xenopus* oocytes as evidenced by its activation by 4αPDD and hypotonicity, its blockage by ruthenium red, and absence from a point mutant that has a disrupted channel filter [19], [20]. All 14 mutants produced large currents that retained the strong outward rectification characteristic of wild type channels (Fig. 1). We have shown previously that this rectification is due to a voltage-dependent gating mechanism distinct from the main intracellular gate, which responds to mechanical and chemical stimulation [19]. This second gating mechanism is apparently not significantly affected by any of the mutations here. Activation upon depolarization and deactivation upon repolarization of the mutants appeared similar to those of the wild type. Whereas wild-type channels partially inactivate during depolarization (Fig. 1, wild type), most mutants, particularly ones that caused more severe clinical phenotypes, lacked such inactivation (*e.g.* compare R616Q and V620I in Fig. 1, which cause the milder BO, to I604M and L618P, which cause neonatal lethal MD). This loss of inactivation is likely a symptom of main-gate malfunction and has been observed in gain-of-function rat-TRPV4 mutants, selected after random mutageneses for their inhibition of yeast growth [19].

**Figure 2:**
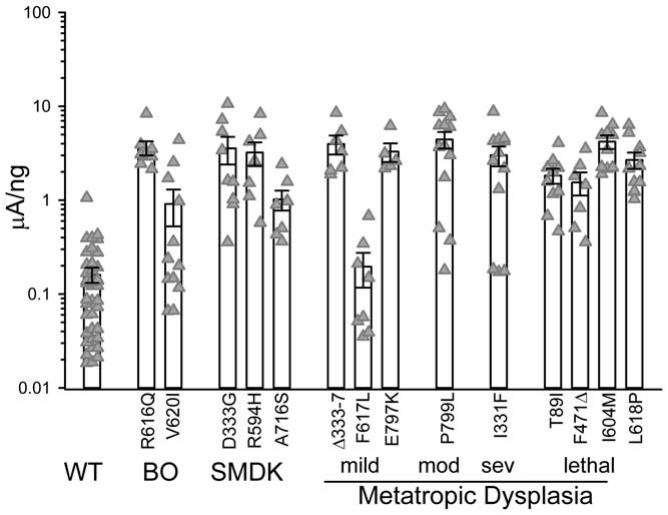
Basal current densities of oocytes expressing *TRPV4* mutant channels are greater than those of wild type but highly variable. Peak currents at +60 mV from oocytes expressing wild type or mutant *TRPV4* assessed between 72 and 96 hours after cRNA injection were measured and standardized to the amount of cRNA injected (triangles plotted on a log scale because of the large range, mean ± s.e.m.). Alleles are grouped by the severity of disease they cause. “WT” denotes wild-type and disease abbreviations are described in the text. MD mutants are further subdivided into mild, moderate (“mod”), severe (“sev”) and infantile/neonatal lethal (“lethal”) as described in Camacho *et al.* (2010) [3]. Whereas the expression of the more severe mutants often reached peak within 24 hours, those of weaker alleles generally took longer and unstimulated wild-type currents were usually not apparent until 72 hours after injection.

Although most mutants generated unstimulated ensemble current levels, normalized to the amount of cRNA injected, larger than those of wild type on average (Fig. 2), we encountered large variations, sometimes of greater than 50 fold (Fig. 2). This large in-group variance makes uncertain the comparisons between mutant groups. Thus, we were unable to distinguish any correlation between the severity of disease and simply the raw mutant basal currents observed here. We have previously observed such variability with TRPV4 expressed in oocytes [20]. Its source could include natural oocyte-to-oocyte variation or uncontrolled variance in the actual amount of full-length cRNA injected. More importantly, we suspect that a negative feedback may limit current density in such a way that toxicity of channel expression triggers a down regulation particularly in the case of the stronger mutants (see Discussion).

### The Loss of Response to Stimulation Parallels the Clinical Severity of the Mutants

**Figure 3:**
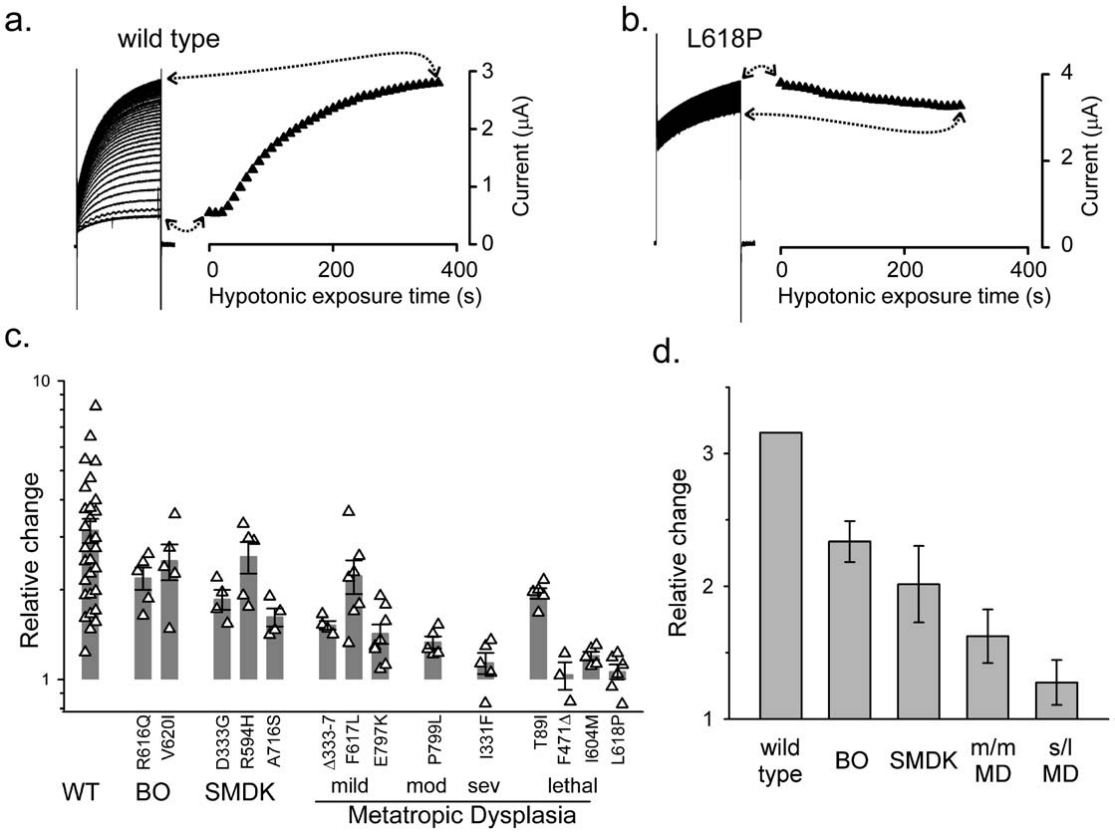
Mutant channels have limited hypotonic responses that inversely correlate with clinical severity. a. Currents were monitored from an oocyte expressing wild-type TRPV4 with 250 ms depolarizations to +40 mV from a hold of 0 mV every 10 s after the removal of 100 mM sorbitol from the media (left, with serial depolarizations vertically stacked). Peak currents from serial depolarizations are plotted against hypotonic exposure time on right. Vertical current scale for both raw traces and plot is shown on right. b. Same as in a, except that oocyte is expressing the neonatal-lethal allele L618P and the test potential was +30 mV. c. Relative increases in peak currents caused by hypotonicity from oocytes expressing wild type and mutant TRPV4 channels assessed at +60 mV. Alleles are grouped by the severity of the dysplasia they cause as described in Fig. 2 legend. Triangles represent individual data points from unique oocytes, vertical bars are the allele means, and error bars are the standard error within each allele. d. Average of the individual allele average hypotonic responses grouped by clinical severity, with error bars representing the s.e.m. of the allele averages. “m/m MD” represents mild and moderate MD, whereas “s/l” represents severe and lethal. Whereas the clinically mildest BO mutants only have a moderate reduction in their response to hypotonicity, most of the severest MD mutants have little or no response.

As a way to sidestep the variation in the *absolute* current magnitude, the *relative* change of each current after stimulation was examined. In contrast to basal current densities, a clear *negative* correlation was apparent between clinical severity and relative response to stimulus of the corresponding mutant channel. A 100 mOsM hypotonic down-shift (350 to 250 mOsm) in the bath caused a saturable increase in wild-type currents over the course of a few minutes (Fig. 3a). This current is reversible upon return to isotonicity and is blocked by ruthenium red [19], [20]. L618P, which causes the most severe neonatal-lethal form of MD, almost completely lacked a response (Fig. 3b). The small decrease here was not consistently observed (Fig. 3c, right). Despite variations, the mutant responses were consistently smaller (Fig. 3c). This decrease clearly correlated with disease severity, particularly apparent when viewed from the perspective of disease-type average (Fig. 3d). Hypotonicity triggered on average a 130% increase in the currents from mutants that cause the mildest form, BO (Fig. 3d). In contrast, it triggered less than a 30% average increase in those that cause the moderate or severe MD (Fig. 3d, right), and most of that increase was due to a single outlier, T89I (Fig. 3c, right group). Of the 4 alleles that cause lethality, F471Δ, I604M and L618P essentially did not respond to hypotonicity (Fig. 3 b, c).

**Figure 4:**
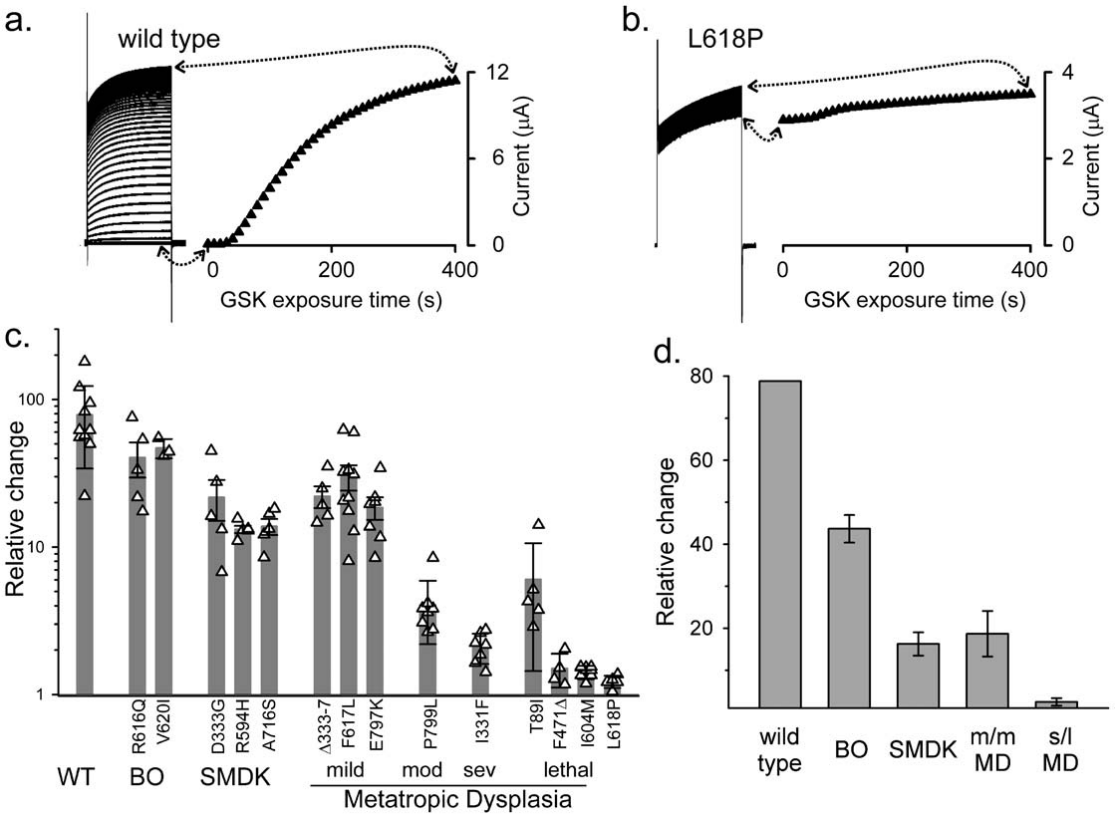
The limitation of the very large GSK responses shows an even more pronounced negative correlation with disease severity. Responses to 1 µM GSK are shown. All figures are arranged as in Fig. 3. Note the near 100-fold increase of the wild-type current here (a, c, d) as compared to the <10-fold increase by hypotonicity. As with hypotonicity, the majority of the most severe mutants have little or no response to GSK (b, c, d).

Even more pronounced is the negative correlation between the clinical severity and the mutant channel's response to a synthetic agonist. GSK1016790A (“GSK”) is a strong TRPV4-specific agonist that is more potent than 4αPDD by several hundred folds [26]. The application of this powerful agonist, unlike the more natural hypotonic stimulus, appear to drive the channel to its activity maximum (See below). Outward currents from oocytes expressing wild-type TRPV4 increase on average 80 fold over the course of a few minutes in response to GSK (Fig. 4a, c left). Again, with the exception of T89I, the lethal alleles had almost no response to even this extremely potent agonist (Fig. 4b, c right group). The mildest BO-inducing mutants gave a ∼50% increase, while the SMDK- and milder-MD-causing mutants gave about 20% increase (Figs. 4c, d). Thus, the reduction in the GSK-induced fraction of the mutant channels robustly parallels the clinical severity of the disease to an even greater extent than in the case of hypotonicity.

### Lack of Stimulus Response is Due to Higher Basal Open Probability

In a previous report [20], we described the single-channel activities of the BO-causing R616Q TRPV4 under patch clamp. R616Q channel retains its conduction, filtration, rectification, and mechanosensitivity property, but has a clearly increase Po even without any stimulation. An increased basal Po leaves less room for its rise to the maximum. This “ceiling” effect seems an obvious reason that underlies the reduced responses of the mutant TRPV4s to hypotonicity or GSK described above (Fig. 3). Although a complete patch-clamp examination of all the mutants is impractical because of the time and labor required, we did test a second and stronger mutant allele: the lethal I604M. The basal Po of wild-type TRPV4 is so low that patches containing only a single activatable channel were essentially silent in the absence of stimulus (Fig. 4a, left). Upon application of GSK, the Po rapidly approached 100% (Fig. 4a, right), consistent with the pronounced 80-fold increase of the wild-type ensemble currents (Fig. 4c, d). In contrast, I604M had a Po near 100% even before GSK exposure and therefore showed only a little increase after (Fig. 4b, note that sporadic spiky closures became less prevalent after treatment). Thus the decrease of the mutants' response to stimuli (Figs. 3, 4) reflects a ceiling effect, not a reduced sensitivity to stimuli. Furthermore, the observation that GSK opened the wild-type channel completely allows inference from the inverse of the ensemble GSK-activated responses (See Discusion).

## Discussion

**Figure 5:**
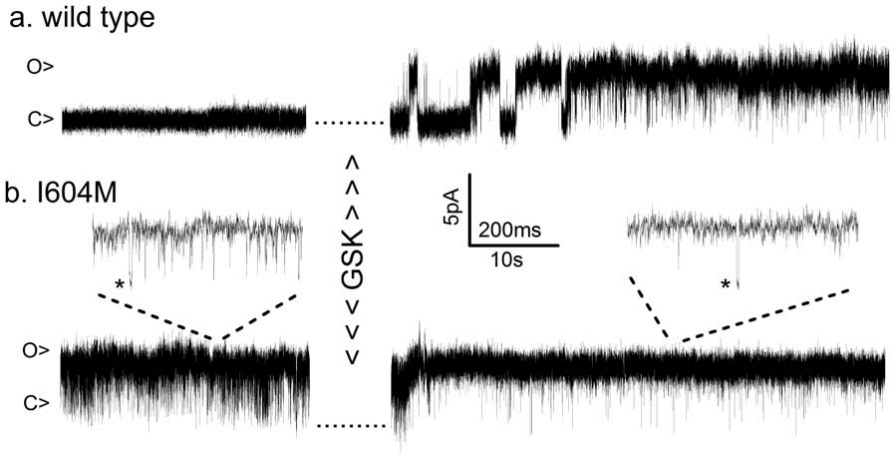
Single channel analysis confirms that lack of response to stimuli is due to saturated basal open probability in I604M. Excised patches with single active channels were analyzed from oocytes expressing either wild-type cRNA (a, n>100) or the neonatal-lethal allele I604M (b, n = 3). Shown are outward unitary currents at +50 mV before (left) and after (right) brief exposure to 1 µM GSK. Open (o) and closed (c) current levels are marked. 50-fold time expansions are shown in B to confirm that the downward spikes indeed reflect the rare full channel closure as evidenced by occasional brief plateaus at the fully closed level (*).

In single-channel analysis, R616Q, which causes the mild brachyolmia, has an increase in basal open probability (Po) compared to wild type [20], but not nearly strong as that of the lethal I604M which has a Po near 1.0 (Fig. 5), directly demonstrating a correlation between basal open probability and resulting clinical severity of the products of these three alleles. Because it is labor intensive to obtain patch-clamp data from all 14 mutants, we deduced the basal Po's of the remaining mutants using two electrode voltage clamp (TEVC). Since little consistent allelic difference was observed in the unstimulated basal currents of oocytes incubated for 3 to 4 days (see a discussion of this below) we instead relied on an analysis of a “ceiling effect” using GSK1016790A. The near lack of response of I604M to GSK in TEVC (Fig. 4) is clearly due to its observed near 100% initial Po (Fig. 5b). The dramatic GSK response of wild-type in TEVC (Fig. 4) clearly results from its low initial Po coupled with GSK's ability to completely open the channel (Fig. 5a) and the reduced response of the milder R616Q (Fig. 4) is consistent with its increased unitary Po observed previously [20]. We are therefore confident that the ceiling effect in the TEVC response to GSK can likewise be used to determine initial unstimulated Po's of the remaining mutants. Another advantage of using the ceiling effect as opposed to basal current densities is that it provides a clear internal control for each oocyte against differences in expression levels, since GSK opens the channels to unity, and therefore basal Po can simply be deduced from the inverse of the relative stimulation by GSK. Such analysis reveals that wild type channels have initial Po's of ∼1% (1/80-fold average stimulation), BO-type channels averaging ∼2% (1/44), SMDK and mild to moderate MD ∼5% (1/18), and severe and lethal MD nearly 40% (1/2.4), with 3 of the 4 lethal alleles having basal Po's near 100%. A robust correlation clearly exists between basal Po and disease severity. The Po increase explains the mutants' dominance over wild type in causing disease as evidenced by the fact that all subjects examined were heterozygous at the *TRPV4* locus.

Because the14 alleles studied here were chosen to represent the distribution of the mutations in the protein and cover the entire SD-disease spectrum, it seems reasonable to assume that all 33 known SD-causing alleles have increased basal open probabilities. Such increases in constitutive activity are well known to result in the “gain-of-function” (GOF) biological phenotypes in other TRP channels. *E.g.* TRPC1 GOF mutations cause retinal degeneration in fly [44], TRPML3 GOFs caused developmental defects of the veritint-waddler mouse [45], [46]. GOFs of TRPV1 [47], TRPV4 [19] and TRPY1 [48] have also been selected through their ability to stop yeast growth. The common mechanism is likely the cytotoxic effect of Ca^2+^ entered through constitutively open channels.

Our conclusion that all SD-causing mutations are GOFs largely agrees with previous findings. Larger ensemble currents were found in 2 BO- [5], 2 SMDK- [4] and 2 MD-causing mutants [3], though not in the SMDK-causing A716S [4]. Here, we found A716S channel to also be overly active (Fig. 1, 2), particularly when assessed by its muted hypotonic or GSK response (Fig. 3, 4). The origin of the discrepancy is unclear and is not easy to sort out because different heterologous expressions (in HEK293 cells *vs.* oocyte) were used. We note that, in the previous study, the Ca^2+^-dependent Fura-2 fluorescence did not increase in the case of R594H and A716S, hinting at a ceiling effect [4], similar to what we observed here.

It is puzzling that a clearer difference between the basal current densities of the BO and the lethal channels shown in Fig. 2 did not stand out against the statistical errors. One simple explanation is that a homeostatic mechanism was at play that down-regulated the stronger GOF channels, possibly by activating a retrieval or degradation pathway in response to deleterious channel activity, perhaps through the Ca^2+^ that leaks in. Consistent with this interpretation, we observed that large current densities of over 5 µA at 60 mV could often be observed from the many mutants after only 24 hours after injection, and current densities generally did not increase beyond 2 days incubation. µA currents from wild-type were never observed before 3 days of incubation despite the fact that its cRNA was injected at ∼10-fold higher concentration (22 ng as opposed to 1–4 ng for the mutants), and current densities increased up to 5 days after injection. In retrospect, if currents were all collected from oocytes expressing TRPV4's for only 24 hours instead of between 72 and 96 hours as had mostly been done in these experiments, then a direct correlation between clinical severity and basal current density may well have been observed. Toxicity of TRPV4 GOF mutations to oocytes has previously been shown [19]. Our experience therefore cautions the use of raw ensemble current alone to assess the severity for the GOF mutants, at least with oocytes.

Our own data are also not without some complexity. Among the neonatal-lethal alleles tested, T89I consistently had a larger response to both hypotonicity and GSK (Figs. 3, 4) than F471Δ, I604M and L618P, the other lethal mutants. While most mutations tested here are predicted to be in the membrane-embedded domain, T89I is at the far N-terminal end, ahead of the ankyrin repeats in the cytoplasm. It seems possible that the pathology T89I induces may include additional mechanisms such as membrane trafficking, beside simple constitutive activities. The GSK responses of the mutants that caused the mildest forms of MD were statistically indistinguishable from those that caused SMDK (Fig. 4d). This does not reflect sampling noise, as evidenced by the fact that E797K and particularly R594H have been recurrently found to cause MD and SMDK respectively, yet have nearly identical GSK responses. Here, disease distinction must be influenced by other facets of TRPV4 activity besides basal current level alone.

The recent finding that R594H can cause the much more severe PD syndrome and that P799L causes SDEM-PM2 has led to the conclusion that the genotype-phenotype correlations are not robust [1]. While there are indeed exceptions, genotypes do appear as a whole to correlate well with disease diagnosis. Although two P799L cases were reported to have SDEM-PM2 [1], the other fourteen P799L cases and four other P799X cases were all diagnosed to have MD [2], [3], [4] and not other forms of dysplasia. Besides the lone case of a single PD patient the remaining sixteen reported R594H cases were all classified as SMDK or SMDK-like [2], [4]. Also, two cases each of F471Δ, R775K, E278K and E797K were consistently classified, as were I133T/F cases [2], [3], [4]. While there is not an absolute correlation, the molecular defects to TRPV4 are clearly the major determinant of disease outcome.

How could the over-activity of TRPV4 lead to developmental defects in skeletal development? TRPV4 participates in the terminal differentiation of developing osteoclasts by providing a sustained Ca^2+^ influx [42]. This Ca^2+^ results the nuclear translocation of NFATc1 (nuclear factor-activated T cell c1), which promotes the expression osteoclast-specific genes, and possibly also in a blockage of apoptosis. As reviewed in the *Introduction*, two concurrent studies indicated that *trpv4^−/−^* mice had decreased osteoclast populations that coincided with increase in bone densities, although this was only observed in response to chronic unloading in one study [39], [42]. If loss of TRPV4 function can lead to a decrease in the bone-absorbing osteoclasts and therefore a subtle increase in bone density, one might predict that GOF mutations could have the opposite effect of increasing osteoclast proliferation during development and resulting in hypoplasic skeletal defects underlying dysplasia, such as wafer-like vertebrae, brachydactyly, delayed carpal ossification, shortened trunk and limbs, facial hyperplasia, *etc*.

Given the dramatic effects GOF mutations has on bone development, it seems paradoxical that *trpv4^−/−^* mice develop normal skeletons at birth [39], [42]. Even the chronically increased bone density, when observed, was not in immature *trpv4^−/−^* mice but only in adults and with no change in overall bone dimension [42]. Therefore, even though TRPV4 is expressed in chondrocytes [43], [49], [50] and osteoclasts, the knock-out phenotypes indicate that TRPV4 channel only plays a redundant, auxiliary, or amplification role in embryonic bone development [39], [42]. While TRPV4 apparently acts in adult unloading-induced osteoporosis [39], this is not a selectable trait during evolution, since animals with weakened bones should have a disadvantage. A tempting speculation is that TRPV4 may instead be involved in an acute bone development response such as fracture repair in the adult. Osteoclasts are known to chemotax to areas of microfracture [51] and play a key role in the final stage of fracture repair, the remodeling of the woven hard callus into the original cortical and/or trabecular bone configuration [52]. TRPV4-mediated osteoclast proliferation may be crucial in this remodeling. TRPV4 activation by mechanical stress could facilitate osteoclast development to the point that the remodeled mature bone relieves this stress. Bone fracture is common in adult vertebrates and early humans. An efficient repair mechanism should be an evolved trait. To our knowledge, fracture healing in *trpv4^−/−^* mice has not been assessed to date.

## Materials and Methods

### Oocyte Expression

Wild-type rat *TRPV4* was integrated into pGH19 vector as described elsewhere [19]. GOF mutations were introduced by a standard 2-step PCR protocol using high-fidelity PfuUltra polymerase (Stragene) and reintegrated into pGH19. cRNA was synthesized from Xho1-linearized templates using a mMessage mMachine T7 kit (Ambion). Stage V and VI *X. laevis* oocytes were each injected with between 1 and 22 ng of cRNA. Channel activities of the stronger GOF mutants appeared and reached near maximum within 24 hours and could be analyzed over the course of several days. Unstimulated wild-type currents were generally not apparent until three days post injection, and the majority of basal and hypotonically stimulated wild-type currents were recorded at or after this point. For the wild-type GSK experiments, it was necessary to use oocytes expressing for only 2 days, since it was critical to only use oocytes with basal currents between 100 and 500 nA at 60 mV, levels high enough to quantitate before GSK exposure but were not subsequently too large for recording conditions after exposure. Since TRPV4 expression, particularly of GOF alleles is toxic to oocytes [19], 1 µM ruthenium red (Sigma) was added to the ND96 incubation buffer. Oocytes were rinsed in ruthenium-free solutions before recording.

### Two Electrode Voltage Clamp (TEVC)

TEVC recording used HS2A head stages and a VG-2Ax100 virtual bath clamp connected to a Gene Clamp 500 amplifier interfaced through a Digidata 1440A digitizer acquired using pClamp10 software (all Axon Instruments). The base bath solution contained (in mM) 66 K^+^-methanosulfate, 100 sorbitol, 1.8 Ba(OH)_2_, and 5 K^+^-HEPES, pH 7.2 (all Sigma). All solutions were maintained under continuous perfusion at a rate of ∼2 mls/min. Sorbitol was omitted from the base solution to form the hypotonic solution. Data was acquired using pClamp10 (Axon Instruments) and analyzed using this as well as Sigma Plot 2000 (SPSS) software and Origin 8.1 (OriginLab). Simultaneously measured membrane voltages showed that they could lag the command voltage by over 5 mV when large currents were maintained. To account for this, linear fits between the measured voltages at +40 and +60 mV commands were always used to extrapolate the values predicted at +60 mV for Figs. 2, 3c and 4c. Leak subtraction was not necessary nor used in any of the TEVC analyses.

### Patch Clamp Analysis

Patch clamp recordings used an L/M EPC7 amplifier (List Medical) interfaced through a Digidata 1440A digitizer using pClamp10 software (both Axon Instruments). Data was acquired at 10 kHz through an eight-pole Bessel filter at 1 kHz, played back at >5 kHz for analysis using pClamp10 software. Borosilicate glass pipettes with ∼1-µm diameter opening at the tip were used. Recordings were done in excised inside-out mode in symmetric (in mM) 98 KCl, 1 MgCl_2_, ad 10 K^+^-HEPES, pH 7.2 (all Sigma). 1 µM GSK was added directly to the bath when tested.
